# Adaptive and dynamic RFID tag anti-collision based on secant iteration

**DOI:** 10.1371/journal.pone.0206741

**Published:** 2018-12-05

**Authors:** Zuliang Wang, Shiqi Huang, Linyan Fan, Ting Zhang, Libin Wang, Yufan Wang

**Affiliations:** 1 College of Information Engineering, Xijing University, Xi’an, Shaanxi Province, China; 2 Microelectronics Research Institute, Hangzhou Dianzi University, Huangzhou, Zhejiang Province, China; 3 Department of Biomedical Data Science, Stanford University, Stanford, California, United States of America; 4 College of Culture Heritage, Northwestern University, Xi’an, Shaanxi Province, China; SPAIN

## Abstract

Radio frequency identification (RFID) has recently experienced unprecedented development. Among many other areas, it has been widely applied in blood station management, automatic supermarket checkout, and logistics. In the application of RFID for large-scale passive tags, tag collision is inevitable owing to the non-cooperation mechanism among tags. Therefore, a tag anti-collision method is a key factor affecting the identification efficiency. In this paper, we propose a tag anti-collision method based on Aloha technology for RFID. It estimates the number of remaining tags using the secant iteration method. To achieve optimal identification efficiency, it adaptively and dynamically adjusts the lengths of the subsequent frames according to the principle that the length of a frame should be the same as the number of tags to be identified. For pseudo-solutions of tag population estimation while using secant iteration, we present an elimination method by two probing frames. The simulation results show that the estimation precision of our method can reach above 97%. Thus, it can meet the requirement of the tag anti-collision estimation accuracy. Its global throughput is obviously superior to the *Q* algorithm adopted by the current international standard, and it is close to the ideal system. It consequently outperforms existing schemes.

## Introduction

Radio frequency identification (RFID) technologies are widely applied in logistics, product tracing, blood management, and many other areas of our daily life [1]. An RFID system consists of readers, electronic tags, and data processing systems [2]. Passive tags are usually attached to the objects to be identified [3]. The data processing systems exchange information through a wireless channel between readers and tags. They then perform the identification, location, and intelligent control of the objects. The quantities of the objects to be identified are usually large. Thus, many tags must often be simultaneously identified. Owing to the cost of that endeavor, tags usually work in passive mode. Consequently, they have neither channel sensing capabilities nor the ability to communicate with each other. Tag collisions may thus occur during batch identification. Tag collisions will result in a decreased identification efficiency and increased time delay, thereby increasing the leakage probability in mobile environments. Therefore, developing a means to reduce the impacts of tag collisions on RFID system identification efficiency is the focus of numerous studies [4–8].

The two types of anti-collision algorithms proposed in the literature are binary-tree-based algorithms [4, 5] and Aloha-protocol-based algorithms [6–8]. In most RFID applications, the tag population size is large. The delay of the binary tree algorithm is too long to meet the requirements of real-time identification in mobile environments. Hence, the Aloha algorithm is widely adopted. It is a random anti-collision algorithm [9, 10] driven by readers, by which each tag randomly transmits in a selected slot according to the reader’s command. This algorithm is simple, and its usage cost is low. The Aloha algorithm is divided into pure Aloha (PA), slot Aloha (SA), frame slot Aloha (FSA), and dynamic FSA (DFSA) [11–13]. The DFSA algorithm was proposed to improve the Aloha performance and is now widely used.

ISO18000 and EPCglobal are the two most influential international standards for RFID. The type C of ISO18000-6 (known as ISO18000-6C) and EPCglobal_C1 G2 (known as EPC_C1 G2) use the same technical system drafted by the EPCglobal organization. The ISO18000-6C standard uses an anti-collision algorithm based on DFSA, and it adopts the *Q* algorithm to dynamically adjust the frame length, frame by frame [6]. The reader sends a command with the frame length *Q* to the tags. Each tag then randomly selects one of the available slots to reply to the reader. This algorithm improves identification efficiency more effectively compared to the fixed frame length method [7–8].

In the Q algorithm, on the other hand, adjustable step *C* only spans from 0.1 to 0.5, and rules of value selection are not provided. In practice, if the value of *C* is too high, the frame lengths must be frequently adjusted and the system will be unstable. On the contrary, if the value of *C* is too low, the adjustment will be sluggish. To address this problem, many improved anti-collision algorithms have been proposed. A method of obtaining high identification efficiency by dynamically adjusting the frame length according to the number of tags was proposed in [14]. In [15], a closed form solution for the analytical calculation of the optimum frame length was presented. Zhang et al. presented in [16] an adaptive assigned tree slotted Aloha protocol that uses a binary query method to solve the unknown tag problem. Kim et al. proposed in [17] a DFSA algorithm that adjusts the frame length based on the number of collision slots and idle slots that engender higher estimation efficiency. The authors of [18] proposed an anti-collision protocol using the reservation mechanism. In this mechanism, the tag identification process is divided into two stages, which reduces the collision slots and eliminates idle slots, greatly enhancing the identification efficiency.

Meanwhile, Bartlett *et al*. proved that the identification efficiency is the highest when the number of tags is the same as the frame length [19]. The authors of [20] presented a tag anti-collision algorithm based on grouping the self-adaptive allocation slot. Accordingly, the system identification efficiency is significantly improved. Shakiba et al. [21] applied the birthday paradox theory to estimate the number of tags. Vogt [22] applied the maximum likelihood estimation method to estimate the number of tags. This approach increases the tag estimation accuracy. Vogt also used the Markov process to describe the dynamic framed ALOHA reading of passive tag.[23] Chen et al. [24] proposed an adaptive algorithm to minimize the total time slots required for identifying the tags within the RFID reader’s interrogation zone. Arjona et al. [25] integrated fuzzy logic with RFID anti-collision protocols, which decreases the identification time by updating the transmission frame size in a dynamic and adaptive way.

For measuring the performance of RFID, global throughput is a commonly used index. It is defined as the ratio of the time consumed by the transmission of information to the time consumed by the whole identification process. Lee et al. [26] presented an algorithm that adaptively updates the frame size based on estimating the number of tags by counting the respective numbers of idle, success, and collision slots. This approach significantly improves the global throughput.

Taking the optimization of global throughput performance as the target, we herein present a FSA-based anti-collision method that can adaptively and dynamically adjust the frame length. It estimates the number of tags to be identified according to the number of tags successfully identified after only the first frame. According to the estimation result and the successfully identified tags in the first frame, the reader adjusts the length of subsequent frames to perform optimal identification. The estimation algorithm adopts the secant iteration. Herein, it is referred to as the secant-iteration-based adaptive and dynamic frame slotted Aloha method (SIADA). The simulation results show that, compared with the *Q* algorithm adopted by the ISO18000-6C standard, the global throughput of the proposed algorithm is improved by more than 2 times. Thus, the proposed method has superior performance.

The remainder of the paper is organized as follows. Section 2 presents the system model and outlines the SIADA algorithm flow. In section 3, we derive a new estimation method of the initial tag population. In section 4, the global throughput of SIADA is presented and compared to the ISO18000-6C *Q* algorithm, the Vogt algorithm, and the ideal system. Finally, concluding remarks are provided in section 5.

## System model

### i. Basic definition

It is assumed that there are multiple passive tags within the reader coverage. The process of completing the identification of all the tags within the reader coverage is called the identification period. One identification period is comprised of multiple identification frames, and each frame is composed of several slots. Within one identification period, each tag only needs to be successfully identified once. When a tag is correctly identified by the reader, it will enter a silent state and will no longer respond to the reader during this identification period.

In addition, it is assumed that there are no new tags entering the reader coverage during one identification period. Suppose there are *n* passive tags within the reader coverage and the identification process is controlled by the reader.

*Query*(*Q*) command: The *Query*(*Q*) command starts a general frame (not including the probing frame). The reader broadcasts the *Query* (*Q*) command to all tags, as the first command to initiate an identification period. It requires the parameter *Q*, whose value is set by the user in advance, and then it is adjusted adaptively and dynamically, according to the number of remaining tags. That is, *Q* refers to the initial value in the first frame, and then iterates from the second frame. All tags enter the active state after receiving this command.

*ReadN* command: A command reads the next slot. 1 is subtracted from *S*_*i*_, *S*_*li*_ or *S*_*ui*_ after the tags receive the *ReadN* command; *S*_*i*_, *S*_*li*_ and *S*_*ui*_ are defined in the next section.

*QueryT* (*Q*) command: The *QueryT* (*Q*) command starts a probing frame. We use two probing frames to eliminate the Pseudo-solution. The details are described in the next section.

### ii. Processing flow

**Fig 1:**
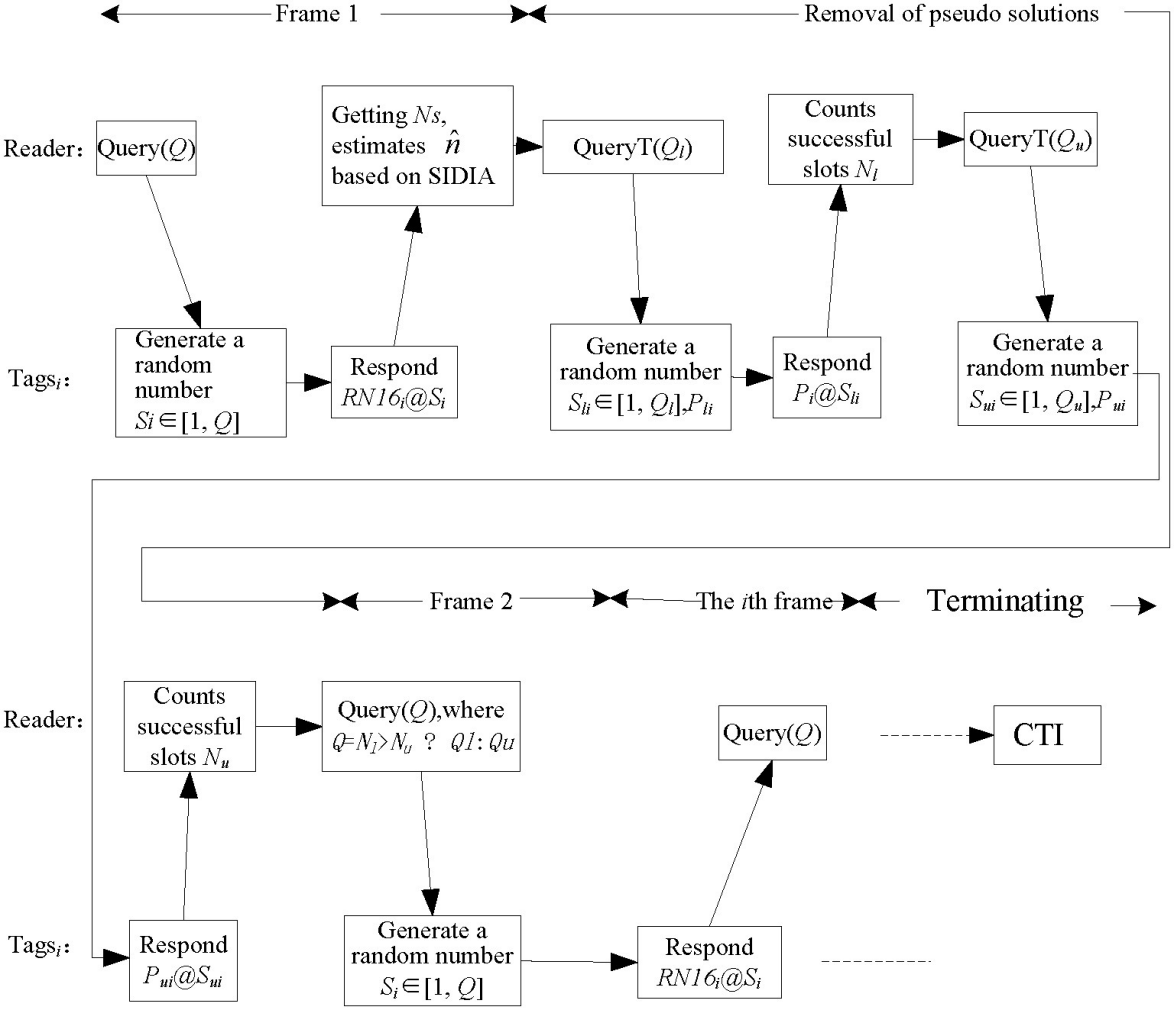
Algorithm processing flow of SIADA (CTI: Criteria of terminating identification).

Firstly, the reader sends the *Query* (*Q*) command, indicating that the frame includes *Q* slots. After receiving the command, each tag randomly produces an integer *S*_*i*_ (*i* = 1, 2…*n*), which is no greater than *Q*. This means that the *i*th tag aims to respond to the reader in slot *S*_*i*_. After that point, the reader sends the command *ReadN*. After receiving *ReadN*, the *i*th tag reduces *S*_*i*_ by 1. If the result is 0, the *i*th tag responds to the reader at this slot; otherwise, it waits for the next *ReadN* command. Accordingly, the reader sends the *ReadN* command for *Q* times to complete the reading of all the *Q* slots in the first frame. For the reader, it does not know the initial number of tags *n*. To enhance the global throughput performance of the overall identification period, it must estimate *n*. In this paper, we propose that, after the successful identification of *N*_*s*_ tags in the first frame, the reader estimates the number of initial tags $\hat{n}$ using secant iteration. Firstly, we set the length of the second frame as ($\hat{n}-N_{s}$) to enable the optimal identification efficiency. Then, the subsequent frames use a similar process until all tags are correctly identified. The algorithm processing flow of SIADA is shown in Fig 1.

As shown in Fig 1, each tag randomly selects one slot to respond to the reader. Slot *S*_*i*_ may be selected by multiple tags, by one tag, or by no tag. If a slot is selected by multiple tags, then there will be more than one tag simultaneously sending its 16-bit random number (RN16) in this slot. The random numbers are different; thus, the reader utilizes the Manchester encoding character to perceive the tag collision. In this case, the slot is marked as a collision slot. If a slot is selected by only one tag, then the slot is an effective slot and *N*_*s*_ is increased by 1. If a slot is selected by no tag, then the slot is an idle slot. Also shown in Fig 1, CTI uses the proposed frame length multiplication algorithm, which is proposed in subsection iii in the next section.

The initial tag population size estimation algorithm is located between the first frame and the second frame, which includes two steps: i.e., initial tag population size estimation based on SIADA, and the removal of the “pseudo solution.” From the second frame, by subtracting the tags that have been successfully identified before, from the initial tags estimated, we can obtain the remaining tags, according to which the length of the current frame will be determined optimally. In this way, the following frames are carried out until all tags are identified successfully. The estimation method consists of solving a transcendental equation, which usually produces two solutions: one of which is a pseudo solution needing to be removed by a special method. The resolvent is given in subsection ii in the next section.

## Estimation of initial tag population

In estimation of the initial tag population, the first frame length is *Q*, and the number of tags to be identified is *n*. Then, each tag randomly generates an integer *S*_*i*_ = *m* (*i* = 1, 2…*n*). The probability that *m* is less than *Q* is *P* = 1/*Q*, and the probability that the *r* tags will simultaneously select one slot is *P*(*Q*, *n*, *r*). Then, [18]

$$P(Q,n,r)=C_{n}^{r}\times {\left(\frac{1}{Q}\right)}^{r}\times {\left(1-\frac{1}{Q}\right)}^{n-r}$$ (1)

Obviously, in accordance with *r* being 1, 0, or greater than 1, the probability of successful, empty, and collision slots can be obtained. When *r* = 1, the probability of successful slots is [18]

$$P(Q,n,1)=(\frac{n}{Q})\times {\left(1-\frac{1}{Q}\right)}^{n-1}$$ (2)

Therefore, the expected value of the number of successful slots in one frame is

$$E[P(Q,n,1)]=Q\times P(Q,n,1)=n\times {\left(1-\frac{1}{Q}\right)}^{n-1}$$ (3)

The left side of (3) is the expected value of the number of tags successfully identified. After the first frame, it can be replaced with *N*_*s*_. We can obtain estimated $\hat{n}$ through solving the equation. Eq (3) is a transcendental equation; we use herein the secant iteration method to solve it.

The Monte Carlo method is used to simulate the global throughput. The Monte Carlo process is outlined as follows. When *n* is given, each tag generates a random number, *S*_*i*_(*i* = 1, 2, 3, …,*n*) ∈ [1, *Q*], representing the ith tag expecting to respond to the reader in slot *S*_*i*_. If *S*_*i*_ = *S*_*j*_(*i* ≠ *j*), then *S*_*i*_ is a collision slot. For any *j* ∈ [1, *n*], if *j* ≠ *i*, then *S*_*i*_ ≠ *S*_*j*_. Hence, *S*_*i*_ is recorded as a successful slot.

### i. Estimation algorithm based on secant iteration

In (3), let *E*[*P*(*Q*, *n*, 1)] = *N*_*s*_, and let

$$f(x)=x{\left(1-\frac{1}{Q}\right)}^{x-1}-N_{s}$$ (4)

By solving the equation *f*(*x*) = 0 and obtaining root *x*, we can then obtain $\hat{n}=[x]$. The secant method is a root-finding algorithm that uses a succession of roots of secant lines to better approximate a root of a function, *f*(*x*). The secant method can be interpreted as a method in which the derivative is replaced by an approximation and is thus a quasi-Newton method. However, Newton’s method requires the evaluation of both *f*(*x*) and its derivative at every step, whereas the secant method only requires the evaluation of *f*(*x*). Therefore, the secant method may be faster in practice. The secant iteration method is based on using the line tangent to the curve of *y* = *f*(*x*), with the point of tangency (*ξ*, *f*(*ξ*)), where *ξ* is the root. Assume that the two initial estimates of the root are *x*_0_ and *x*_1_. Then, the tangent function is

g(x)=ax+b (5)

with *g*(*x*_0_) = *f*(*x*_0_), *g*(*x*_1_) = *f*(*x*_1_).

This line is called the secant line. Its equation is derived as

$$g(x)=\frac{\left(x_{1}-x)f(x_{0})+(x-x_{0})f(x_{1}\right)}{x_{1}-x_{0}}$$ (6)

and is linear in *x*. Solve the equation *g*(*x*) = 0, and denote the root by *x*_2_. This yields

$$x_{2}=x_{1}-\frac{f(x_{1})(x_{1}-x_{0})}{f(x_{1})-f(x_{0})}.$$ (7)

The same process is repeated. Use *x*_1_ and *x*_2_ to produce another secant line, and then use its root to approximate *ξ*. This yields the general iteration formula

$$x_{n+1}=x_{n}-\frac{f(x_{n})(x_{n}-x_{n-1})}{f(x_{n})-f(x_{n-1})}\;n=1,2,3\ldots$$ (8)

To start the secant iteration, three parameters, namely *x*_0_, *x*_1_, and *N*, must be specified, where *N* is the maximum number of iterations. Assume that *ε* is the error tolerance. The stopping criterion is that |*f*(*x*_*n*+1_)| ≤ *ε*, |*x*_*n*+1_ − *x*_*n*_| ≤ *ε*, or the iteration times ≥ N. The secant iteration algorithm is shown in Table 1.

**Table 1:** Secant iteration algorithm.

Step 0: Initial setting. Set *N*, *x*_0_, *x*_1_, count = 0.
Step 1: Compute $y_{0}=x_{0}{\left(1-1/Q\right)}^{x_{0}-1}-N_{s}$ and $y_{1}=x_{1}{\left(1-1/Q\right)}^{x_{1}-1}-N_{s}$.
Step 2: Iterating, *x* = *x*_1_ –*f*(*x*_1_)(*x*_1_ –*x*_0_)/(*f*(*x*_1_) − *f*(*x*_0_)), *x*_0_ = *x*_1_, *x*_1_ = *x*.
Step 3: *if* |*f*(*x*_*n*+1_)| ≤ *ε*, *|x*_*n*+1_ *− x*_*n*_*| ≤ ε*, or *k* ≥ *N*, go to step 1, else stop.

*Count* represents the number of iterations. Input: *x*_0_, *x*_1_ and *N*. Output: *x*

### ii. Pseudo-solution removal

Calculate the first-order derivative of Eq (4). Then, we can obtain

$$f\prime (x)={\left(1-\frac{1}{Q}\right)}^{x-1}[1+x\;\text{ln}(1-\frac{1}{Q})]$$ (9)

In a real application system, *Q* >> 1; thus, (1 − 1/*Q*)^*x*−1^ > 0. Using the Taylor series expansion equation, we can obtain

$$f\prime (x)\approx {\left(1-\frac{1}{Q}\right)}^{x-1}(1-\frac{1}{Q}x)$$ (10)

Let Eq (10) be equal to 0. We can obtain the extreme point, *x*_*opt*_≈*Q*. That is, when the number of tags to be identified is equal to *Q*, the identification efficiency reaches the maximum. Theoretically, *N*_*s*_ should not be greater than $x_{opt}{\left(1-1/Q\right)}^{x_{opt}-1}$. When $N_{s}=x_{opt}{\left(1-1/Q\right)}^{x_{opt}-1}$, Eq (4) has the sole solution. According to Eq (10), it can be observed that, when *x*<*Q*, then *f*’(*x*) > 0; thus *f*(*x*) monotonically increases. When *x*>*Q*, then *f*’(*x*) < 0, and *f*(*x*) monotonically decreases. Therefore, when $N_{s}<x_{opt}{\left(1-1/Q\right)}^{x_{opt}-1}$, there are two solutions to Eq (4), which are respectively located at each side of *x*_*opt*_. One of them must be a pseudo-solution and should be eliminated.

Two initial values, *x*_0_ and *x*_1_, should be set to solve Eq (4) when the secant iteration is adopted. *Q* is known. Therefore, in the case of double solutions, the solution position can be controlled through the initial value setting. By setting *x*_0_ and *x*_1_ to be smaller than *Q*, the solution *x*_*l*_ will be smaller than *x*_*opt*_. Otherwise, if setting *x*_0_ and *x*_1_ to be greater than *Q*, then the solution *x*_u_ will be greater than *x*_*opt*_.

Setting $Q_{l}={\hat{n}}_{l}-N_{s}$ and $Q_{u}={\hat{n}}_{u}-N_{s}$ respectively, the reader sends two probing commands *QueryT*(*Q*_*l*_) and *QueryT*(*Q*_*u*_) in two identification probing frames. Where, ${\hat{n}}_{l}=round(x_{l})$ and ${\hat{n}}_{u}=round(x_{u})$. The effectively identified tag numbers, *N*_*sl*_ (corresponding to $Q_{l}={\hat{n}}_{l}-N_{s}$) and *N*_*su*_ (corresponding to $Q_{u}={\hat{n}}_{u}-N_{s}$), are recorded. Those tags that are successfully identified during the statistical stage will not enter silent states. The reader determines the one that is a pseudo-solution according to the identification efficiency. If $N_{sl}/({\hat{n}}_{l}-N_{s})\geq N_{su}/({\hat{n}}_{u}-N_{s})$, then $\hat{n}=[x_{l}]$; otherwise, $\hat{n}=[x_{u}]$. After eliminating the pseudo-solution, the following frames will continue until all tags are effectively identified. Then, the identification period will be finished.

Table 2 gives the experimental results of the estimation algorithm based on the secant iteration. The experimental parameters are *n* = 900, *Q* = 1200, *ε* = 0.001; *x*_0_ = 0.3Q = 360 and *x*_1_ = 0.6Q = 720 for estimation of *x*_l_, and *x*_0_ = 1.3Q = 1560 and *x*_1_ = 1.6Q = 1920 for estimation of *x*_u_.

**Table 2:** Experimental results of estimation algorithm based on secant iteration.

*x*	*x*_0_	*x*_1_	*E*[*P*(*Q*, *n*, 1)]	*N*_*s*_	*x*_*n*_	Iterations
*x*_*l*_	360	720	425.4	428	*x*_*0*_ = 360, *x*_*1*_ = 720, *x*_*2*_ = 811 *x*_*3*_ = 890, *x*_*4*_ = 917, *x*_*5*_ = 923	4
*x*_*u*_	1560	1920	425.4	420	*x*_*0*_ = 1560, *x*_*1*_ = 1920, *x*_*2*_ = 1610, *x*_*3*_ = 1619, *x*_*4*_ = 1620, *x*_*5*_ = 1620	4

Table 2 shows that the number of iterations of solving *x*_*l*_ and *x*_*u*_ is four. By utilizing the experimental results of Table 2, the reader sends two probing commands *QueryT*(495) and *QueryT*(1200) based on the parameters *Q*_*l*_ = 923−428 = 495 and *Q*_*u*_ = 1620–420 = 1200. Through the experiment, we obtain *N*_*sl*_ = 189, with 189/495 = 0.3816, and *N*_*su*_ = 315, with 315/1200 = 0.2624. Owing to 0.3816>0.2624, we can obtain $\hat{n}=923$ and the estimated error is |923–900|/900 = 2.5%. Such estimation accuracy can meet the requirement of the frame length adjustment. If utilizing the theoretical value *E*[*P*(*Q*, *n*, 1)] = 425.4 for estimation, the experiment result is *x*_*l*_ = 897 and *x*_*u*_ = 1563. Therefore, *N*_*sl*_ = 172, with 172/472 = 0.3640, and *N*_*su*_ = 312, with 312/1138 = 0.2739. After eliminating the pseudo-solution by utilizing the same method, we can obtain $\hat{n}=897$, and the estimated error is |897–900|/900 = 0.3%. It can be observed that the estimation error is mainly derived from the deviation between *N*_s_ and the theoretical value *E*[*P*(*Q*, *n*, 1)]. If these two values are equal, the estimation error of the initial number of tags will be quite small.

### iii. Criteria of terminating identification

According to the definition in this paper, the identification period is the complete identification of all tags in the reader’s coverage. It is therefore necessary to determine whether all tags have been successfully identified in order to determine whether to terminate the identification period. Furthermore, the DFSA protocol suffers the well-known tag-starvation problem; that is, a tag may not be correctly read during a reading cycle. Thus, the criteria of terminating identification play a key role in the identification cycle.

The SIADA method proposed in this paper does not require counting the empty slots and collision slots. In theory, it can decide whether to terminate the identification period only according to the successfully identified tags within the frame. If the number of successfully identified tags is 0, we can assume that all tags have been successfully identified. However, through the simulation experiment, it is found that this method is likely to be wrong. In the *Query*(*Q*) command, parameter *Q* is equal to the value that the initial tag number estimated after the first frame minus the number of all tags successfully identified before this frame. Accordingly, the first frame estimation error will pass to the last frame. The estimation accuracy of the SIADA algorithm is higher than 97%. When the remaining tag population is large, it is effective to adaptively adjust the frame length according to the estimated number of the remaining tags. However, when the population is small, such as less than ten, if the number of initial tags is approximately thousands, the estimated error will be greater than a few dozen, which is greater than the number of remaining tags. It is possible that the estimated number of remaining tags will be 0. Nevertheless, the actual number of tags is not 0. To solve this problem, the following frame length multiplication algorithm is proposed.

Assuming that Rep is a natural number, which is the upper-bound of cycles, then the termination judgment is as follows.

Step 1: *Q*_*i*_ = *Q*_*i*-1_-*N*_s*i*-1_.
Step 2: If *Q*_*i*_ = 0 and the number of cycles is less than Rep, then *Q*_*i*_ = 2*(*Q*_*i*_+1), broadcast the *Query*(*Q*_*i*_) command, and proceed to Step 1. If *Q*_*i*_ = 0 and the number of cycles is equal to Rep, then proceed to Step 4. Otherwise, proceed to Step 3.
Step 3: Start a new identification frame.
Step 4: End the identification period.

If the parameters are properly configured, the frame length multiplication algorithm can solve the tag starvation problem.

The procedure of SIADA, where *n* is equal to 200, 400, 800 and 2000, is simulated, and the results are shown in Table 3. The other parameters: Rep = 3; *ε* = 0.001; *x*_0_ = 0.3*Q* and *x*_1_ = 0.6*Q* for estimation of *x*_l_; *x*_0_ = 1.3*Q* and *x*_1_ = 1.6*Q* for estimation of *x*_u_.

**Table 3:** The procedure experiment of SIADA.

Frame	The number of remaining tags after each frame											
	*n* = 200, *Q* = 300 *Ns* = 103,${\hat{n}}_{l}=201$ ${\hat{n}}_{u}=427$,$\hat{n}=201$		*n* = 400, *Q* = 600 *Ns* = 201,${\hat{n}}_{l}=391$ ${\hat{n}}_{u}=872$,$\hat{n}=391$		*n* = 800, *Q* = 1200 *Ns* = 412,${\hat{n}}_{l}=806$ ${\hat{n}}_{u}=1704$,$\hat{n}=806$		*n* = 800, *Q* = 500 *Ns* = 162,${\hat{n}}_{l}=287$ ${\hat{n}}_{u}=797$,$\hat{n}=797$		*n* = 2000, *Q* = 3000 *Ns* = 1026,${\hat{n}}_{l}=1994$ ${\hat{n}}_{u}=4297$,$\hat{n}=1994$		*n* = 2000, *Q* = 1400 *Ns* = 479,${\hat{n}}_{l}=931$ ${\hat{n}}_{u}=2004$,$\hat{n}=2004$	
	NA	NE	NA	NE	NA	NE	NA	NE	NA	NE	NA	NE
1	97	98	196	187	388	394	638	635	974	968	1521	1525
2	63	64	133	124	234	240	393	390	625	619	933	937
3	40	41	80	71	138	144	251	248	405	399	607	611
4	25	26	57	48	79	85	153	150	257	251	363	367
5	15	16	36	27	44	50	104	101	162	156	226	230
6	10	11	26	17	21	27	77	74	101	95	152	156
7	5	6	19	10	10	16	45	42	64	58	93	97
8	4	5	15	6	-	-	26	23	42	36	58	62
9	2	3	8	7	-	-	21	18	23	17	34	38
10	-	1	7	6	-	-	16	13	14	8	18	22
11	-	-	4	3	-	-	11	8	12	6	7	11
12	-	-	2	1	-	-	9	6	10	4	4	8
13	-	-	-	2	-	-	5	2	8	2	2	6
14	-	-	-	-	-	-	4	5	5	3	-	4
15	-	-	-	-	-	-	2	3	3	1	-	-
16	-	-	-	-	-	-	-	1	-	1	-	-

(NA: Number of Actual-remaining tags; NE: Number of Estimated- remaining tags)

Table 3 shows that, when *n* = 200 and *Q* = 300, 10 frames are required to identify all tags; when *n* = 400 and *Q* = 600, 12 frames are required; when *n* = 800 and *Q* = 1200, 7 frames are necessary; when *n* = 800 and *Q* = 500, the requirement is 16 frames; when *n* = 2000 and *Q* = 3000, 16 frames are necessary; when *n* = 2000 and *Q* = 1400, 14 frames are needed. Table 3 also shows that when (*n*, *Q*) = (200,300), (800,1200) and (2000,1400), with the numbers of estimated-remaining tags being more than the numbers of actual-remaining tags, the “multiplication frame length algorithm” is not enabled. In the other three cases, the numbers of estimated-remaining tags are less than those of actual-remaining tags, so the “multiplication frame length algorithm” is enabled.

## Results and discussion

### i. Estimation performance of SIADA

We compare the estimation performance with two estimators that have been proposed in references [23] and [27], respectively. They are widely used in applications. Zhen *et al*. propose in [27] that the number of initial tags can be approximately estimated as follows:

$$\hat{n}=N_{s}+2.39N_{c}.$$ (11)

where, *Nc* denotes the number of collision slots. Vogt *et al*. propose in [23] that a lower bound on the value of *n* can be obtained by the simple estimation function *ε*_*lb*_, which is defined as *ε*_*lb*_ = *N*_*s*_ + 2*N*_*c*_.

**Fig 2:**
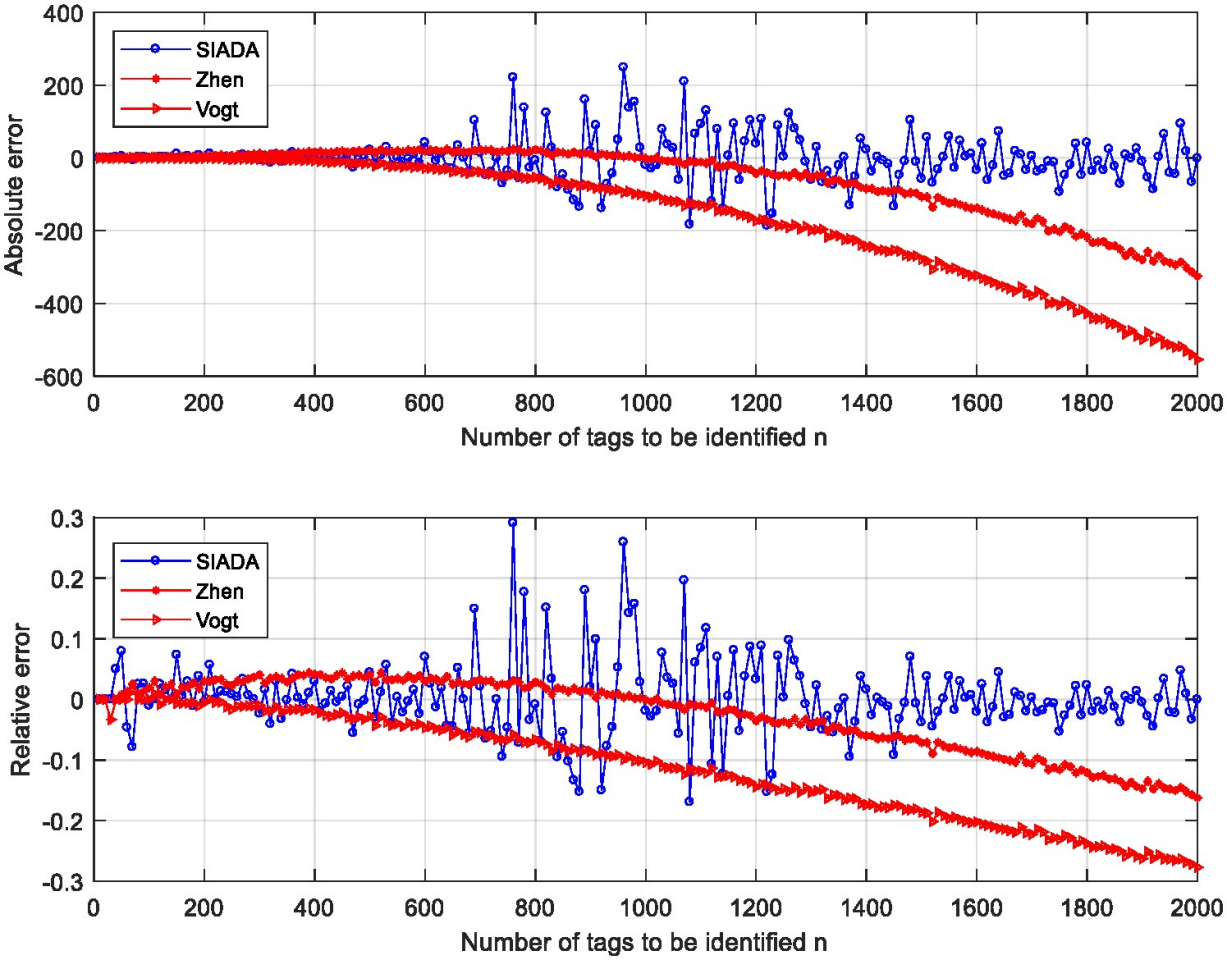
Estimation performance of SIADA and the other two estimators.

We use Matlab to simulate the SIADA-based estimation of the number of initial tags and compare it with Zhen algorithm and Vogt algorithm. The simulation results are shown in Fig 2. The simulation parameters are *Q* = 1000, *ε* = 0.01, *n* is from 10 to 2000, the sampling interval is 10, and the number of performed trials is 100.

Fig 2 demonstrates the absolute error and relative error of SIADA, Zhen, and Vogt algorithm. We can see that SIADA outperforms Zhen and Vogt algorithm. Fig 2 also shows that when *n* is less than 700 or more than 1300 the relative error of SIADA is less than the relative error of Zhen and Vogt algorithm. When *n* is close to *Q*, i.e., it is approximately between 800 and 1200, the relative error of SIADA is slightly larger. This finding shows that, when the number of tags to be identified is close to *Q*, which is near the optimal value of identification, the method of pseudo-solution removal is prone to error. In this case, the error of pseudo-solution removal will engender a certain degree of influence on the tag identification performance. Nonetheless, owing to the difference between the two roots, *x*_*l*_ and *x*_*u*_, being insignificant, it will not cause the algorithm to fail. On the other hand, when *n* is far from *Q*, the estimation error is quite small. This is because, when *n* is close to *Q*, the two solutions, *x*_*l*_ and *x*_*u*_, located at each side of *x*_*opt*_, are close to each other. Owing to the inherent randomness of *N*_*s*_, some errors will inevitably occur during the elimination of the pseudo-solution. Fortunately, in this case, the difference between solutions *x*_*l*_ and *x*_*u*_ is small. Thus, the false elimination of pseudo-solutions will not significantly increase the estimation error, and it will not have a fatal impact on the SIADA identification performance.

### ii. Global throughput comparison

The global throughput is a commonly used index for measuring the performance of RFID. Specifically, it is defined as:

$$\eta_{syn}=\frac{time\;consumed\;by\;transmission\;information\;}{time\;consumed\;by\;the\;identification\;period\;}.$$ (12)

The time consumed by the identification period is the sum of the transmission time for all commands, interval time between commands, state switching time, time associated with successful, idle and collision slots, and estimation time. It is assumed that the length of each slot is the same.

Assume the command codes of *Query*(*Q*), *QueryT*(*Q*), and *ReadN* as in Table 4.

**Table 4:** Command Codes.

Command Name	Command Code (2 bits)	Parameter(12 bits)
*Query*(*Q*)	00	XXXXXXXXXXXX
*QueryT*(*Q*)	11	XXXXXXXXXXXX
*ReadN*	01	---------

*RT*_*cal*_ is defined as Reader-to-Tag calibration symbol and *TR*_*cal*_ as Tag-to-Reader calibration symbol. According to the EPCglobal_C1 G2 standard, the *TRcal* and *RTcal* must meet the constraints in Eq (13):

$$1.1\times RT_{cal}\leq TR_{cal}\leq 3\times RT_{cal}.$$ (13)

The length of information sent to the reader by the tags is defined as *L* bits. R-to-T (Reader to Tag) data rate *R*_*rt*_ is assumed to be equal to 26.7 kbps, *L* is equal to 16, and *TR*_*cal*_ = 1.2 × *RT*_*cal*_. T-to-R (Reader to Tag) data rate *R*_*tr*_ is equal to *R*_*rt*_ /1.2 = 22.25 kbps. A Reader shall set *RT*_*cal*_ equal to the length of a data-0 symbol plus the length of a data-1 symbol (*RT*_*cal*_ = 0_length_+1_*length*_). Thus, *RT*_*cal*_ = 74.9μs (assuming equiprobable data) and *TR*_*cal*_ = 89.9 μs (assuming equiprobable data). The divide ratio is defined as DR. A DR is assumed equal to 64/3(EPC standard). Backscatter-link pulse-repetition interval is defined as *T*_*pri*_. *T*_*pri*_ = *TR*_*cal*_ /DR = 4.2μs.

Assume the time from *Query*(*Q*) (or *ReadN*) to tag response as *T*_1_. According to EPCglobal_C1 G2 standard, *T*_1_ = *Max* (10*T*_*pri*_, *TR*_*cal*_). Thus, *T*_1_ = 89.9 μs. The time from tag response to *Query*(*Q*) (or *ReadN*) is defined as *T*_*2*_. According to EPCglobal_C1 G2 standard, *T*_*2*_ shall meet the constraint: 3*T*_*pri*_ ≤ *T*_2_ ≤ 20*T*_pri_. So, *T*_*2*_ = 5*T*_*pri*_ = 21 μs is assumed.

In Table 3, with *n* = 800 and *Q* = 500 as an example, it takes 17 frames and 2219 slots to identify 800 tags. The Reader broadcasts 17 *Query*(*Q*)s, 2 *QueryT*(*Q*)s and 2219 *ReadNs*. The lengths of both *Query*(*Q*) and *QueryT*(*Q*) are 14. The length of *ReadN* is 2. From Table 3, we can get that *Ns* = 162, ${\hat{n}}_{l}=287$, and ${\hat{n}}_{u}=797$. *Q*_*l*_ = 287–162 = 125 and *Q*_*u*_ = 797–162 = 635. The length of the estimation slots is 760. It is assumed that a tag responds to the reader with 4 bits data and not RN16 after *QueryT*(*Q*) to shorten the delay. The estimation time is 760*4*0.5*89.9 = 136648μs. The time consumed by the identification period is 17*14*0.5*74.9+2*14*0.5*74.9+ 2219*16*0.5*89.9+760*0.5*4*89.9+2219*89.9+2219*21 = 1988600μs. The time consumed by transmission information is 800*16*0.5*89.9 = 575360 μs. So, *η*_*syn*_ = 575360/1988600 = 0.29. In our experiments, the command transmission time is 8913.1 μs. The time associated with slots is 1978648 μs. 8913.1 ≪ 1978648. For simplicity, only the numbers of successful, idle, collision, and estimation slots, are counted in our simulation. The state switching time highly depends on the device performance in applications. As it is difficult to measure the state switching time, we ignored it in the simulation of this paper.

To objectively and accurately evaluate the performance of our proposed anti-collision algorithm, we provide the simulation results of global throughput. We employ our estimator, a Zhen estimator, and an ideal system respectively in our procedure. In the ideal system, the number of initial tags is known in advance. We also give the throughput of the classic DFSA algorithm used in ISO18000-6C.

In the *i*th frame, [27] proposes that the *a posteriori* probability distribution of *k* tags choosing the slot is

$$p_{k}^{0}(i)=\{\begin{array}{ll} 0 & if\;k=0.1\\ \frac{p_{k}(i)}{1-p_{0}(i)-p_{1}(i)} & if\;k\geq 2 \end{array}$$ (14)

In other words, the *a posteriori* expected value of the number of tags is respectively, 0 for an empty slot, 1 for a success slot, and $\sum_{k=2}^{n}kp_{k}^{0}(i)$ tags for a collision slot. Therefore, the estimated tag sets in the current frame is $p_{1}(i)+\sum_{k=2}^{n}kp_{k}^{0}(i)$. The number of initial tags can be approximately estimated with Eq (11).

The parameter *Q* of SIADA, ideal system and Zhen system, is the frame length, however, in ISO18000-6C, the frame length is expressed by 2^*Q*^, *Q* here is the base 2 logarithm of frame length.

**Fig 3:**
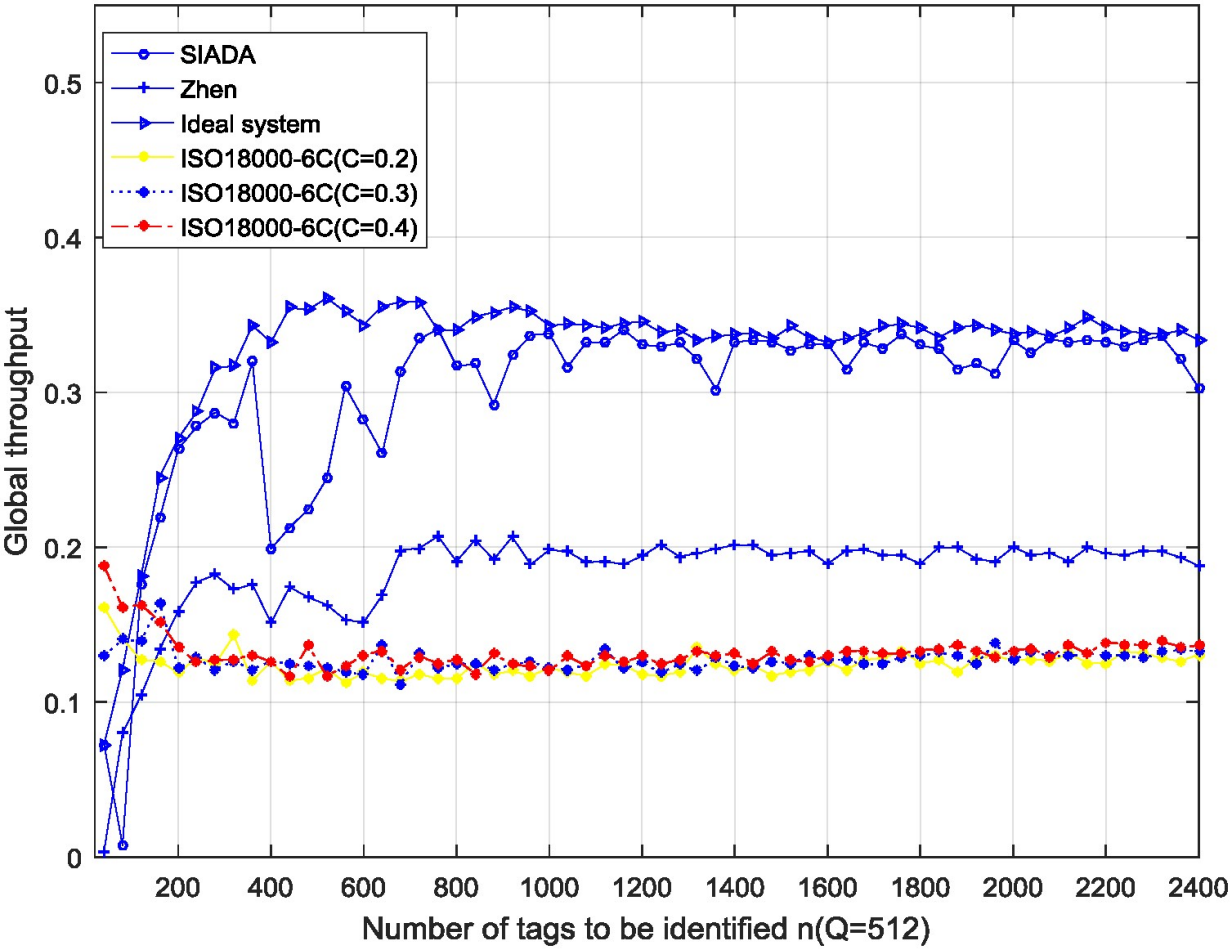
Simulation results of global throughput performance; *Q* = 512 (*Q* = 9 for ISO18000-6C).

**Fig 4:**
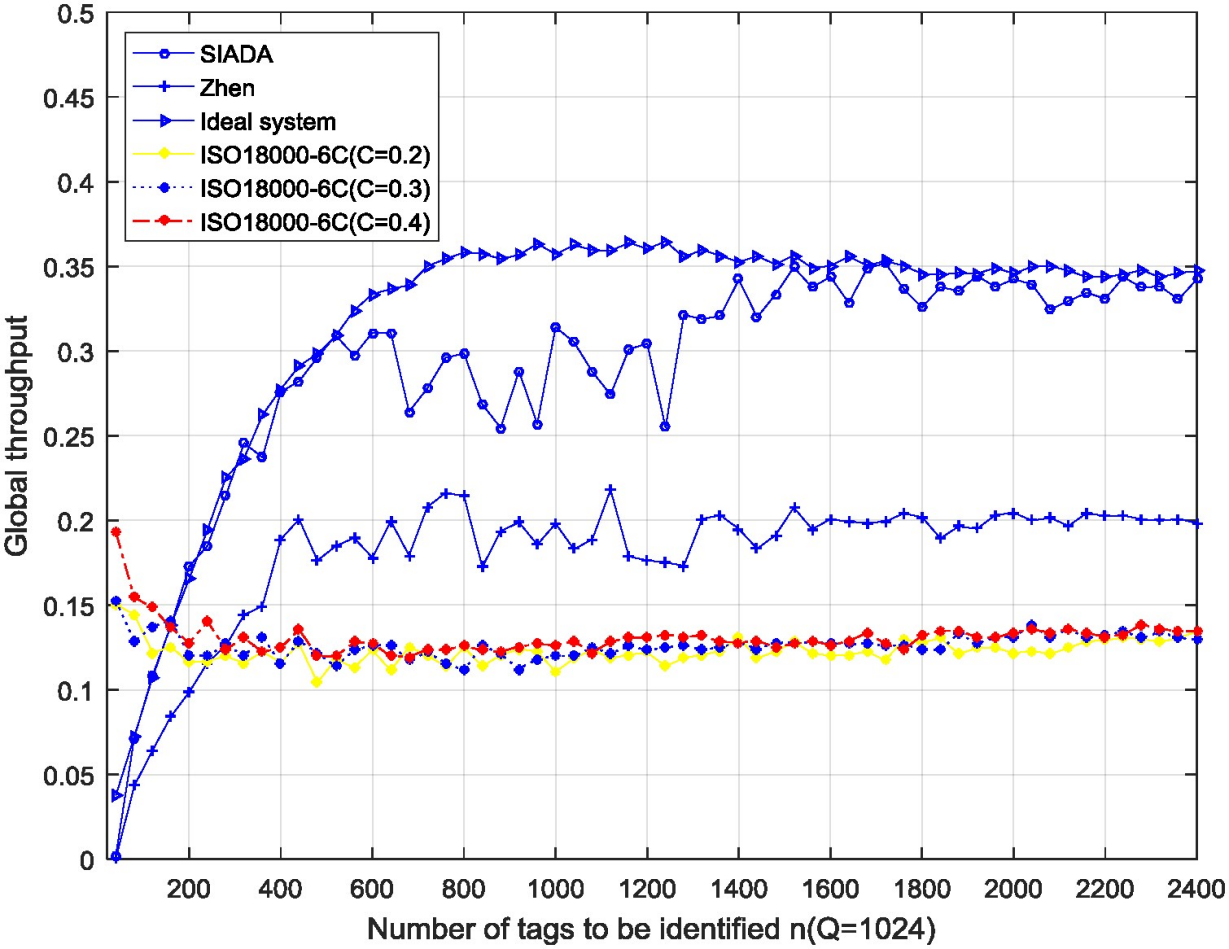
Simulation results of global throughput performance; *Q* = 1024 (*Q* = 10 for ISO18000-6C).

**Fig 5:**
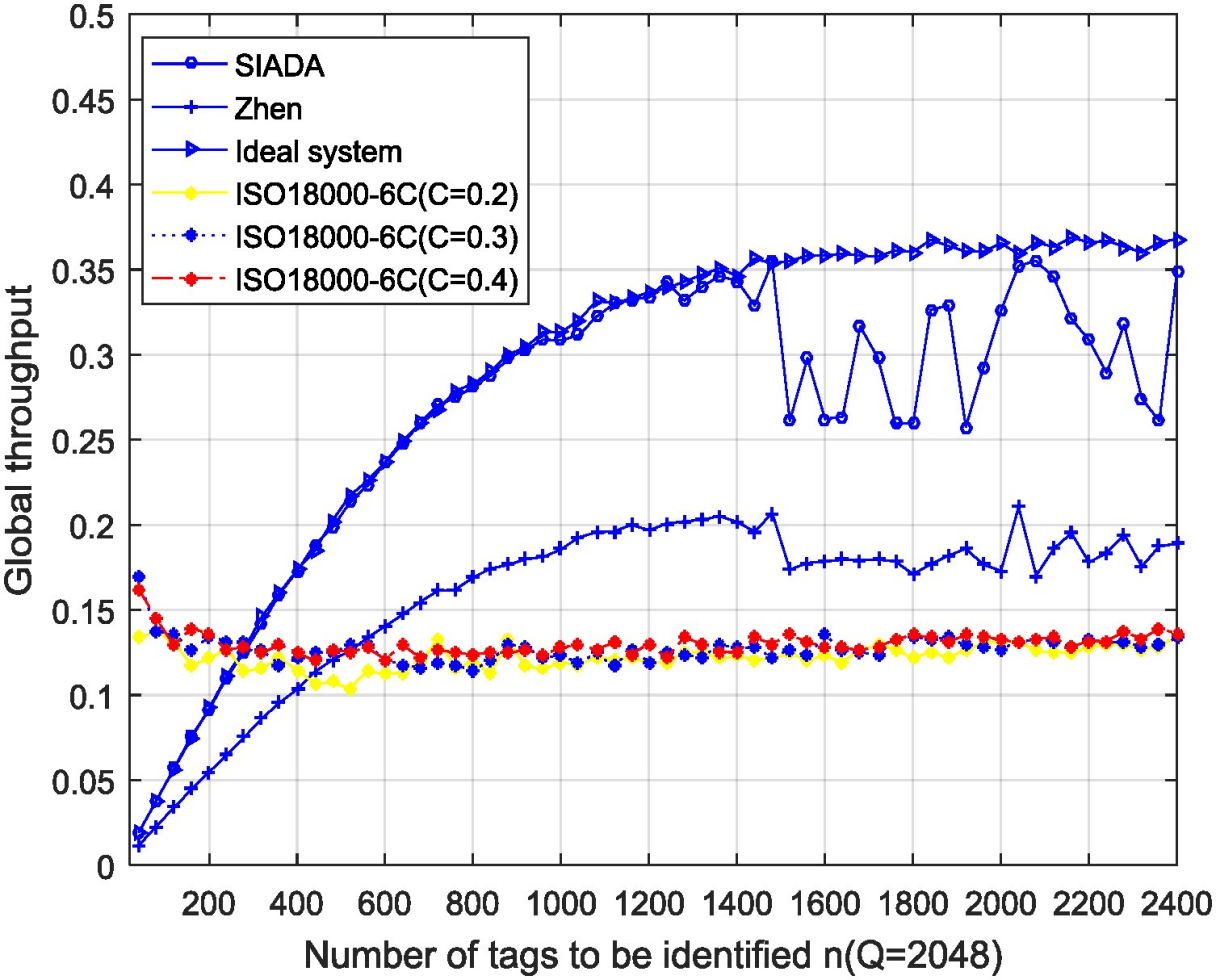
Simulation results of global throughput performance; *Q* = 2048 (*Q* = 11 for ISO18000-6C).

The results are shown in Figs 3, 4 and 5. The simulation parameters are *ε* = 0.01, *n* is from 40 to 2400, the sampling interval is 40, Rep = 3, and 100 trials are conducted.

In the ideal system, the initial number of tags (*n*) is known in advance, so the number of remaining tags is also known. The initial frame size is still *Q* (512, 1024 or 2048 in the experiments), but from the second frame, the frame size (*Q*) is adjusted accurately to be equal to the number of remaining tags. Then, it adaptively and dynamically adjusts the frame length to strictly comply with the optimal identification standard, that is, the frame length should be equal to the number of remaining tags. ISO18000-6C adopts the *Q* algorithm given in appendix *D* of [6] to adjust the frame length. Here, step *C* = 0.2, 0.3 and 0.4 respectively. The other main simulation parameters are *Q* = 512, 1024, and 2048, respectively. (In the ISO18000-6C standard, the frame length is expressed by 2^*Q*^; thus, *Q* is actually the base 2 logarithm of the frame length. Hence, *Q* = 9, 10, and 11, respectively). In addition, *ε* = 0.01, and *n* is from 40 to 2400 with the step 40.

From Figs 3, 4 and 5, when the number of tags to be identified is about greater than 100, the SIADA global throughput performance is significantly better than that of the *Q* algorithm. When the number of tags to be identified is near *Q* (such as when *Q* = 1024, *n* varying from 700 to 1300), the global throughput performance of SIADA decreases. The lowest value is 0.26, which is approximately twice that of ISO18000-6C and 75% of the ideal system. When *n* is far from *Q*, the performance approximates the ideal system, which is about twice more than that of ISO18000-6C.

The throughput is not very stable when *n* is near *Q*. This is because, when the number of tags to be identified is close to the initial frame length, the two solutions, *x*_*l*_ and *x*_*u*_, located at each side of *x*_*opt*_, are close to each other, which causes the pseudo-solution removal algorithm to tend to fail more frequently. This results in adjusting the *Q*-value at a non-optimal frame length, and it has a certain impact on the global throughput performance. Nevertheless, even if the global throughput of SIADA decreases to the lowest point 0.17 when the tag number is 800, it is still far greater than the *Q*-algorithm of the standard EPC_C1 G2.

For the *Q*-algorithm, the throughput is always between 0.1 and 0.14, even if the initial *Q*-value is not perfectly matched to the actual population size. This is because the *Q*-algorithm adjusts the frame length by multiplying by 2 or dividing by 2, so that the convergence rate is very fast. Thus, the initial *Q*-value has no obvious effect on the global throughput performance. This conclusion is consistent with the results of existing literature which hold that the number of tags has a minimal impact on the performance. We additionally find that parameter *C* has no significant impact on the throughput performance.

### iii. Comparison of delay

Given the initial number of tags *n*, by defining the time required to successfully identify all *n* tags as *delay*(*n*), we can obtain

$$delay(n)=\frac{L*n/R_{tr}}{\eta_{syn}}.$$ (15)

**Fig 6:**
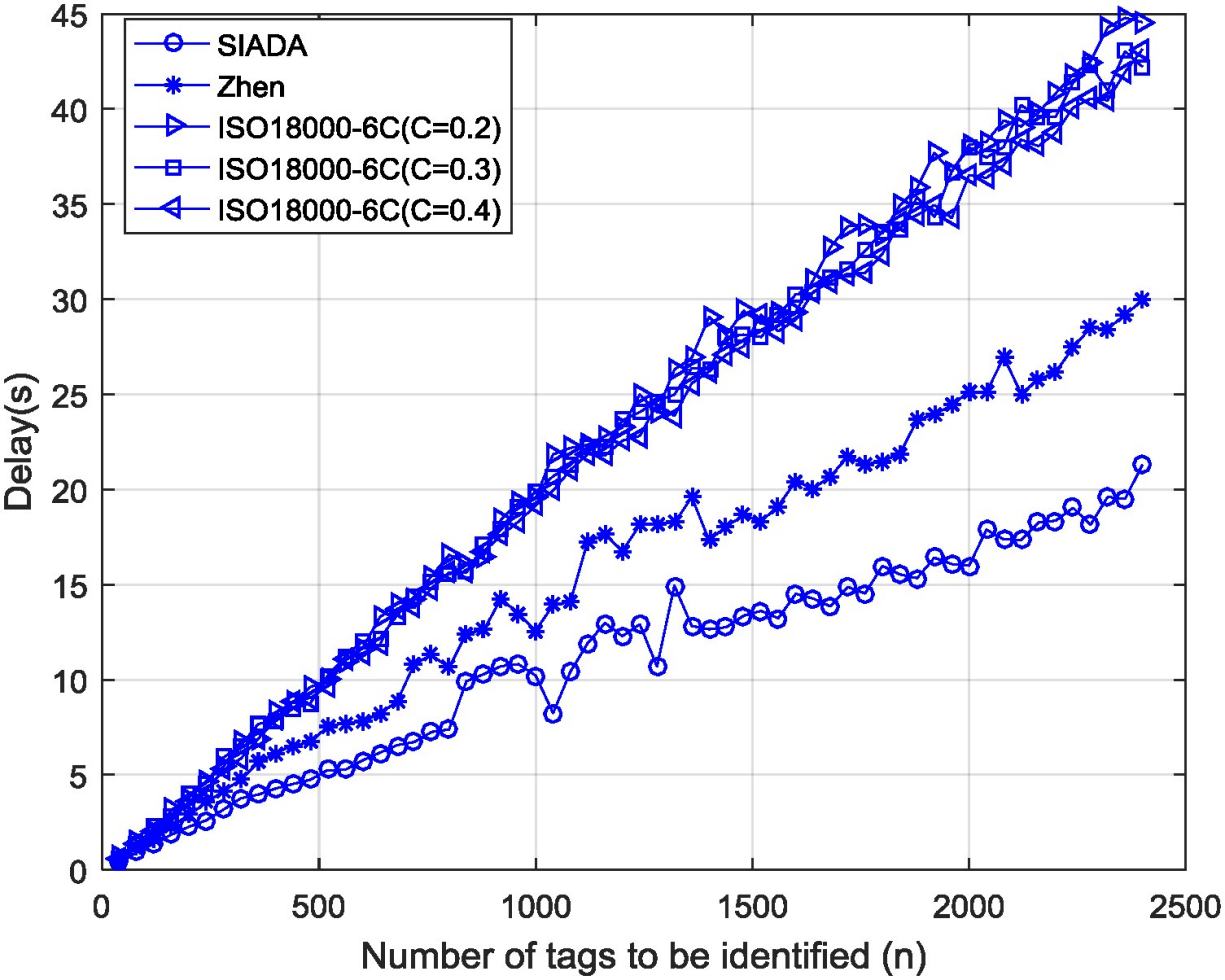
Delay results of SIADA, Vogt and ISO1800-6C.

According to the EPCglobal_C1 G2 standard, assume the R-to-T data rate *R*_*rt*_ is equal to 26.7 kbps, the T-to-R data rate *R*_*tr*_ is equal to 22.25 kbps and *L* is equal to 16. The delay results are shown in Fig 6.

It can be observed in Fig 6 that the delay of the SIADA algorithm is the smallest, the ISO1800-6C delays are the largest, and the Vogt algorithm delay is somewhere between them. When *n*>500, the SIADA delay is approximately 50% less than that of ISO1800-6C, and approximately 20% less than that of Vogt.

## Conclusion

Compared with the traditional DFSA, the new anti-collision algorithm proposed in this paper provides a significant improvement in the global throughput performance, which is very close to the ideal system. Owing to a paper length constraint, we herein did not address the balance between the processing delay and identification efficiency in the case of a large number of tags. In real applications, it can be solved by appropriate grouping of tags.

## Supporting information

S1 Dataset (ZIP)
